# Photoacoustic imaging of the dynamics of a dye-labeled IgG4 monoclonal antibody in subcutaneous tissue reveals a transient decrease in murine blood oxygenation under anesthesia

**DOI:** 10.1117/1.JBO.28.11.116002

**Published:** 2023-11-30

**Authors:** Anjul Khadria, Chad D. Paavola, Konstantin Maslov, Patricia L. Brown-Augsburger, Patrick F. Grealish, Emmanuel Lozano, Ross L. Blankenship, Rui Cao, Junhui Shi, John M. Beals, Sunday S. Oladipupo, Lihong V. Wang

**Affiliations:** a California Institute of Technology, Caltech Optical Imaging Laboratory, Andrew and Peggy Cherng Department of Medical Engineering, Pasadena, California, United States; b Eli Lilly and Company, Lilly Corporate Center, Lilly Research Laboratories, Indianapolis, Indiana, United States; c Eli Lilly and Company, Lilly Biotechnology Center, Lilly Research Laboratories, San Diego, California, United States; d California Institute of Technology, Caltech Optical Imaging Laboratory, Department of Electrical Engineering, Pasadena, California, United States

**Keywords:** monoclonal antibody, antibody imaging, blood oxygen, photoacoustic imaging

## Abstract

**Significance:**

Over 100 monoclonal antibodies have been approved by the U.S. Food and Drug Administration (FDA) for clinical use; however, a paucity of knowledge exists regarding the injection site behavior of these formulated therapeutics, particularly the effect of antibody, formulation, and tissue at the injection site. A deeper understanding of antibody behavior at the injection site, especially on blood oxygenation through imaging, will help design improved versions of the therapeutics for a wide range of diseases.

**Aim:**

The aim of this research is to understand the dynamics of monoclonal antibodies at the injection site as well as how the antibody itself affects the functional characteristics of the injection site [e.g., blood oxygen saturation (${\mathrm{sO}}_{2}$)].

**Approach:**

We employed triple-wavelength equipped functional photoacoustic imaging to study the dynamics of dye-labeled and unlabeled monoclonal antibodies at the site of injection in a mouse ear. We injected a near-infrared dye-labeled (and unlabeled) human IgG4 isotype control antibody into the subcutaneous space in mouse ears to analyze the injection site dynamics and quantify molecular movement, as well as its effect on local hemodynamics.

**Results:**

We performed pharmacokinetic studies of the antibody in different regions of the mouse body to show that dye labeling does not alter the pharmacokinetic characteristics of the antibody and that mouse ear is a viable model for these initial studies. We explored the movement of the antibody in the interstitial space to show that the bolus area grows by ∼300% over 24 h. We discovered that injection of the antibody transiently reduces the local ${\mathrm{sO}}_{2}$ levels in mice after prolonged anesthesia without affecting the total hemoglobin content and oxygen extraction fraction.

**Conclusions:**

This finding on local oxygen saturation opens a new avenue of study on the functional effects of monoclonal antibody injections. We also show the suitability of the mouse ear model to study antibody dynamics through high-resolution imaging techniques. We quantified the movement of antibodies at the injection site caused by the interstitial fluid, which could be helpful for designing antibodies with tailored absorption speeds in the future.

## 1 Introduction

Monoclonal immunoglobulin G (IgG) antibody-based drugs have provided life-saving options for people with various diseases, including COVID-19.^1^^,^^2^ Over the last four decades, over 100 monoclonal antibodies have received FDA approval to treat several disorders, with a preponderance of them approved in the previous decade.^3^^,^^4^ Despite being extensively utilized as therapeutics, limited knowledge exists regarding their basic behavior at the injection site; thus, an opportunity exists to establish baseline information to advance molecule selection^5^^,^^6^ and subcutaneous formulation development. Following subcutaneous injections, monoclonal antibodies are slowly absorbed via the lymphatic system due to their large hydrodynamic size; however, the formulation excipients can diffuse away from the injection site rapidly via absorption across the microvasculature.^7^^,^^8^ The slow absorption of the antibodies from the injection site via the lymphatic system extends the residence time at the injection site; thus, the behavior of these molecules is ultimately affected by the local conditions of the injection site, including the pH, temperature, architecture of extracellular matrix, viscosity of the interstitial fluid, and muscle movement.^9^^–^^12^ Apart from being affected by the injection site conditions, the antibodies themselves can influence changes in tissue characteristics, such as temporary hypersensitivity reactions.^13^^,^^14^ The study of changes in local hemodynamic features, such as oxygen saturation (oxygenation), oxygen extraction fraction (OEF), and change in total hemoglobin, could provide insights into unknown effects of antibodies at the injection site. Apart from increasing use as drug therapeutics, monoclonal antibodies conjugated with contrast agents have found increased use for visualization of different structures in the body through different imaging modalities.^15^^–^^17^ Thus, the study of the effects of antibodies on the injection site characteristics may further help in designing safer and more effective therapies and reagents for medical use.

Here, we use optical-resolution photoacoustic microscopy (OR-PAM) to study the subcutaneous injection site dynamics of the near-infrared (NIR) dye-labeled human IgG4 isotype control antibody, as well as its effects on the local hemodynamics at the injection site of mice ears. We selected the IgG4 isotype control antibody with amino acid substitutions to minimize the effector function as a molecular tool for this work because of its lack of target mediated interaction at the site of injection, favorable biophysical properties, and ready availability. Although the variable domains of this antibody do not have any specific paratope, the constant domain of the antibody is still able to engage in binding to FcRn receptors during pinocytosis.^18^^,^^19^

## 2 Materials and Methods

### 2.1 Preparation of Sulfo-Cyanine-7.5 Conjugated IgG4 Antibody

The sulfo-cy7.5 dye-labeled IgG4 antibody was prepared and characterized as previously reported.^17^ An IgG4 isotype antibody was conjugated with sulfo-cyanine-7.5 dye via NHS ester chemistry (66320, Lumiprobe). The antibody was prepared at a concentration of ∼10  mg/mL in a buffer comprising 90 mM carbonate, 9 mM phosphate, and 125 mM sodium chloride, adjusted to a pH of 8.3. The dye was dissolved in a carbonate buffer, occupying 1/10th of its volume immediately before being added to the antibody, ensuring a 10-fold excess of dye, and then was subjected to a 4-h incubation at 25°C. Excess dye was separated from the labeled antibody using size exclusion chromatography on a Superdex S200 column (Cytiva) with a mobile phase consisting of 1× phosphate-buffered saline (PBS) at pH 7.2, flowing at a rate of 1  mL/min. The degree of labeling was determined through MALDI-MS analysis, revealing ∼4.5  dye molecules per antibody molecule. Subsequently, the antibody was concentrated to ∼30  mg/mL using a 15 mL spin concentrator (Millipore) equipped with a 100 kDa molecular weight cutoff membrane. Samples of both labeled and unlabeled antibodies (5 to 10  μg) were subjected to high-performance liquid chromatography (HPLC) employing an Agilent 1260 Infinity II system and an Agilent AdvanceBio SEC 300 Å 2.7 mm column (PL1580-3301, Agilent). The HPLC was conducted at a flow rate of 1  mL/min, with a mobile phase consisting of 1× PBS at pH 7.2 (20012-027, GIBCO), and peaks were detected through absorbance measurements at 214 nm. The entire chromatographic run was completed within 7 min. The molar absorption spectra of the dye-labeled antibody are given in Fig. S1 in the Supplemental Material.

### 2.2 Optical Resolution Photoacoustic Microscopy System Design

We used a ring-shaped transducer (central frequency = 42 MHz, f-number = 1.67, Capistrano Labs) in the OR-PAM setup, which is equipped with three lasers of 532, 559, and 780 nm optical wavelengths. Light pulses of all three wavelengths were used to shine (532 and 559 nm at 80 nJ, and 780 nm at 200 nJ) at the same point successively with microseconds delay. The delay between pulses with different wavelengths was chosen as a tradeoff with two conditions: (1) delays should be as short as possible to ensure that the PA signal is generated at the same or nearly the same sample voxel and (2) delays should be long enough for photoacoustic signals from different pulses to be separable in the time domain. The light beams from a 559 Nd:YAG laser (BX2II, Edgewave) and 532 nm light (SPOT-10-200-532, Elforlight) laser were combined using a polarization beam splitter (PBS121, Thorlabs) and focused on a 254  μm diameter orifice (3928T991, McMaster-Carr) for spatial filtering. The 780 nm light was emitted from a dye (Styryl 11 dye in 200 proof ethanol, 07980, Exciton) laser (Credo, Sirah) pumped by a 532 nm Nd:YAG laser (IS80-2-L, Edgewave GmbH). The 780 nm beam was spatially filtered by focusing it on a second pinhole (3928T991, McMaster-Carr). The 532 and 559 nm light beams were combined with the 780 nm light beam through a dichroic mirror (M254C45, Thorlabs). The combined light beam was focused on the sample through an achromatic doublet (AC080-020-A, Thorlabs) after collecting some amount of light by a photodiode (PDA36A, Thorlabs) through a beam sampler to correct for laser fluctuations. The mouse was scanned using a two-dimensional (2D) scanner built with stepper motors (PLS-85, Physik Instrumente) that were controlled by a customized LabVIEW program with an FPGA (PCIe-7841, National Instruments). All of the data were acquired through a digitizer (ATS 9350, AlazarTech) at a frequency of 500 MS/s.

### 2.3 Animal Experiments

#### 2.3.1 Imaging experiments

We performed all imaging experiments on animals using protocols approved by IACUC at Caltech. We used Hsd:Athymic Nude-Fox1nu mice aged 6 to 12 weeks (Envigo) in the photoacoustic experiments while maintaining their body temperatures at 37°C during imaging. The mice were acclimated for at least 4 days before the imaging experiments. Before the mouse was imaged for the first time, we put a black sticker on its ear punched with a hole of dimension ∼3  mm×6  mm. The black sticker acted as a guide or marker for us to position its ear under the microscope at the same place every time before imaging. Mice were imaged under isoflurane anesthesia (1.25% to 1.50% isoflurane in the air at a flow rate of 1  L/min) as well as ketamine as per the requirement of the experiments. For experiments in which anesthesia was performed under ketamine, the mouse was injected with a 0.4 mL of ketamine/xylazine cocktail before the imaging was started, and then 0.1 mL of the same cocktail was injected every 45 min until 180 min post injection of the dye-labeled IgG4 antibody. The ketamine/xylazine cocktail was formed by diluting 0.225 mL of 100  mg/mL of ketamine and 0.125 mL of 20  mg/mL xylazine in 2.15 mL of saline.

#### 2.3.2 Pharmacokinetic studies of human IgG4 and sulfo-cy7.5 labeled human IgG4 antibodies

Mouse pharmacokinetic study protocols were approved by the IACUC at Eli Lilly and Company. Female CD-1 mice (6 to 12 weeks old, ∼20 to 35 g in weight) were obtained from Envigo (Indianapolis, Indiana). Animals were acclimated for at least 4 days before test article administration. The unlabeled IgG4 and the sulfo-cy7.5 dye-labeled IgG4 antibody samples were formulated at 0.02  mg/mL for intravenous (IV) and torso subcutaneous (SC) dosing or 20  mg/mL for SC ear dosing in PBS, pH 7.2. Mice were anesthetized using isoflurane by inhalation of ∼2% in the air. A volume of 200 mL of the 0.02  mg/mL antibody was delivered by the IV (tail vein) or SC (torso) route (0.004  mg/mouse). For SC administration in the ear, a volume of 0.2  μL was administered using a 2.5  μl glass syringe (600 Series, Hamilton) with a removable needle assembly (7632-01; Hamilton) affixed with a 34-gauge, 0.5-in. needle (207434; Hamilton) (0.004  mg/mouse). Sample collection was performed by making sequential tail nicks at 1, 2, 6, 24, 48, 72, 96, 120, 168, 336, and 504 h after test article administration. 10  μL of whole blood was collected with a micro-capillary (Drummond Scientific), and the sample was then immediately dispensed into 90  μL of Reagent E buffer (Gyros, Uppsala Sweden). The resulting 100  μL sample was centrifuged (2000-RCF, 10 min), after which the supernatant was transferred to a labeled polypropylene cluster tube and stored at −70°C before analyses. Human IgG concentrations were determined in the whole blood samples performed on the Gyros xPand instrument using the Gyros Generic hIgG pharmacokinetic kit (P0020499). The standard curve range was from 2 to 2000  ng/mL for IgG or 5 to 5000  ng/mL for sulfo-cy7.5 dye-labeled IgG4.

Standard curve regression was performed on Gyros Evaluator regression software to interpolate unknown sample concentrations using a five-parameter logistic fit model of the fluorescence responses at the 1%-photomultiplier tube (PMT) setting (Gyrolab User Guide, 2018; section D, Data Analysis, Chapter 4, p. D-33)

The concentrations determined based on the 10% mouse blood/90% Rexxip A buffer samples were transformed to plasma concentrations by multiplying by a factor of 17.36. The correction factor accounts for mouse hematocrit and dilution effects during the whole-blood sample processing.^20^

Plasma pharmacokinetic parameters were determined using Phoenix WinNonLin version 8.1.0.3530. Values below the quantification limits were ignored in the pharmacokinetic parameter calculations.

### 2.4 Imaging Protocol

We adapted an imaging protocol as previously reported.^17^

The murine ear was imaged with a rapid A-line rate of 4 kHz using a fast axis (2.5  μm step size, 1100 steps) and a slow axis (5  μm step size, 800 steps), totaling 220 s of scanning. For imaging the dye-labeled IgG4 antibody formulations in the mouse ear, we first acquired a baseline image, followed by a sub-microliter injection of the antibody. Subsequent images were taken at 3, 15, 30, 60, and 180 min post-injection while the mouse remained anesthetized. The time-point for each image was considered the midpoint of the acquisition. After 180 min, the mouse was awakened, provided food and water, and re-imaged at the 6- and 24-h time points post-injection. Individual experiments with different time points are mentioned specifically in the paper.

### 2.5 Calculating the Area Occupied by the Dye-Labeled IgG Antibody Bolus to Study Its Movement

Maximum amplitude projection (MAP) images of the sulfo-cy7.5 dye-labeled IgG4 antibody (0.1  μL, 20  mg/mL, n=3) were acquired from the raw photoacoustic data. The MAP images were thresholded (after passing through a median filter of size 4×4  pixels) by the summation of the mean and three times the standard deviation of the background amplitude to segregate photoacoustic signals (generated by the dye-labeled antibody) from the noise in the region of interest. The total number of pixels within the thresholded region of interest was multiplied by the size of a single pixel (2.5  μm×5.0  μm) to calculate the area occupied by the antibody bolus. The area occupied at each time point was divided by the area at 3 min (just after injection) to calculate the normalized area.

## 3 Results and Discussion

### 3.1 Injection Site Dynamics of Antibody

**Fig. 1:**
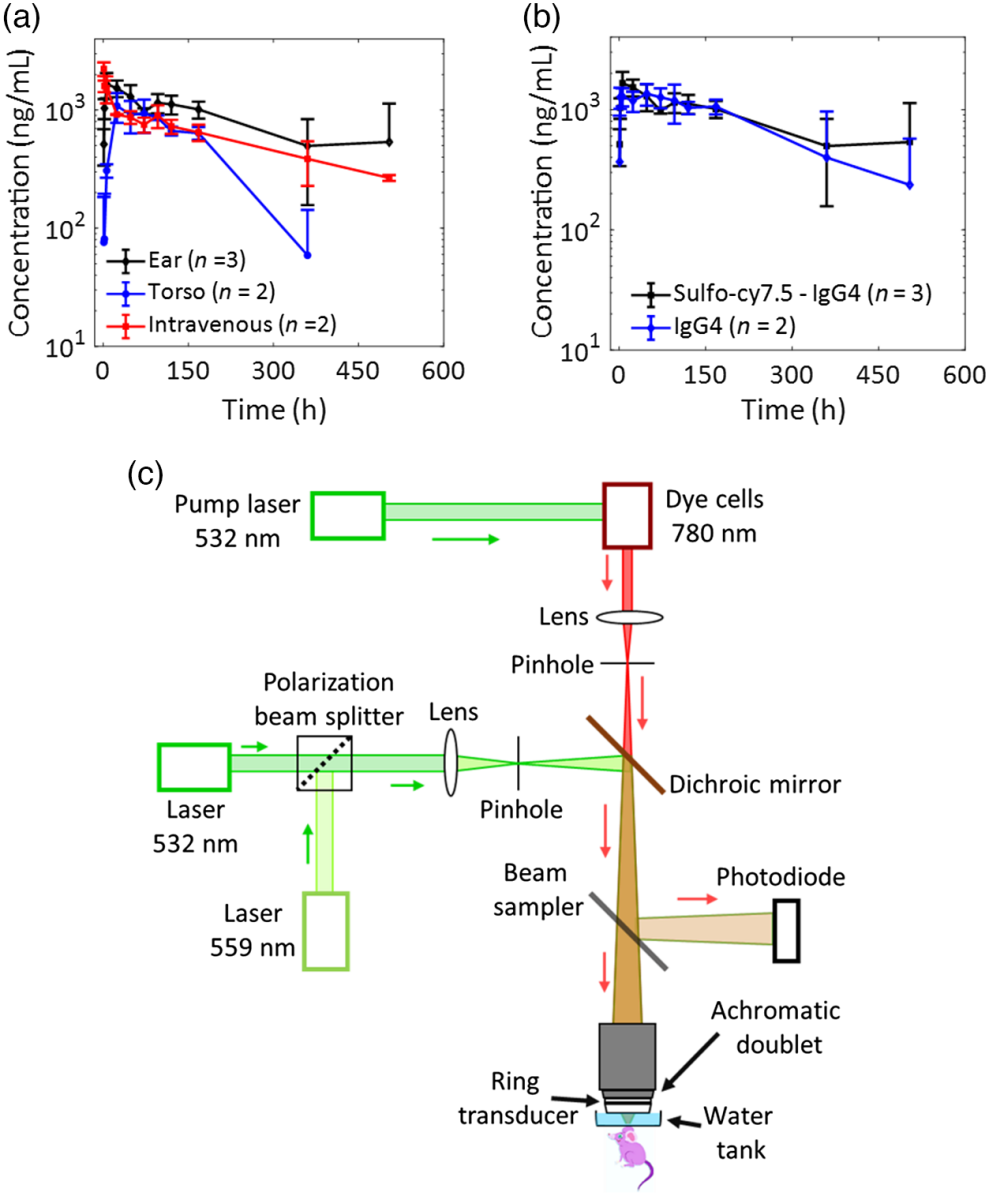
(a) Comparison of bioavailability of the dye-labeled antibody through different routes of injections in mice. (b) Comparison of dye-labeled and unlabeled antibody solutions dosed subcutaneously in mice ears shows that the dye labeling does not alter the pharmacokinetics of the antibody. (c) Schematic of the triple-wavelength equipped OR-PAM. All data represent mean ± standard deviation.

We labeled the IgG4 antibody with the sulfo-cy7.5 dye through a previously described method.^17^ Before analyzing the absorption behavior of the dye-labeled IgG4 antibody by photoacoustic imaging, we first investigated the suitability of the mouse ear model for this study, as well as the effects of labeling on the pharmacokinetics of the antibody. Dye-labeled and unlabeled IgG4 isotype control antibody solutions were injected into the mice by the intravenous route and by subcutaneous injections in the torso or ear. Pharmacokinetic blood samples were collected over 504 h (21 days) following administration [Fig. 1(a) and Fig. S2(a) in the Supplemental Material]. We observed similar pharmacokinetic profiles for both subcutaneous locations, thus confirming that the mouse ear model is suitable for our study. We also demonstrated that sulfo-cy7.5 dye labeling did not appreciably alter the pharmacokinetic properties of the antibody by comparing the pharmacokinetic profiles of the dye-labeled and unlabeled antibody solutions in the mouse ear [Fig. 1(b) and Tables S1 and S2 in the Supplemental Material].

We designed and employed a triple-wavelength equipped OR-PAM [Fig. 1(c)] to study the NIR light absorbing sulfo-cy7.5 dye-labeled IgG4 isotype control antibody in the mouse ear. Our initial efforts to quantify the injection site absorption kinetics of the dye-labeled IgG4 antibody through photoacoustic imaging using the previously reported method were unsuccessful [Figs. S2(b) and S2(c) in the Supplemental Material].^21^ The results suggested that <25% of the dye-labeled antibody disappeared from the injection site after the first 3 h; however, at the 6 and 24 h time points, the total photoacoustic signal was higher than the total signal just after injection (i.e., at 3 min), which could not be the true reflection of the injection site kinetics of the antibody. The increase in the total photoacoustic signal was hypothesized to be the result of changes in the environment of the dye-labeled antibody, which led to a dynamic change in its molar absorption coefficient. In such a scenario, a change in photoacoustic signals cannot be directly considered to be the change in the concentration of dye-labeled antibodies.^22^ The antibody solution was prepared in PBS, which can be readily absorbed across the microvasculature, whereas the hydrodynamically large antibody is predominantly absorbed via the lymphatic vessels at a concomitantly slower rate due to diffusion and slow drainage of the lymphatic fluids.^7^^,^^23^ Because the salts in the PBS vehicle are absorbed at a different rate than the antibody, the changing solution conditions in the injection site result in dynamic shifts in the molar absorption coefficient of the dye-labeled antibody, making quantification studies unreliable.

Pharmacokinetic quantification of dye-labeled antibodies prepared in buffer solutions using fluorescence microscopy has been reported several times.^24^^–^^26^ However, due to the dynamic change of the molar absorption coefficient as a result of different absorption mechanisms of the formulation components and the antibody, most of the studies have observed an increase in fluorescence, complicating the pharmacokinetic analysis. As a negative control, we quantified the absorption kinetics of the sulfo-cy7.5 dye alone, dissolved in the PBS buffer, and observed that most of the dye (∼90%) disappears from the injection site within the first 3 h of injection [Fig. S2(d) in the Supplemental Material].

**Fig. 2:**
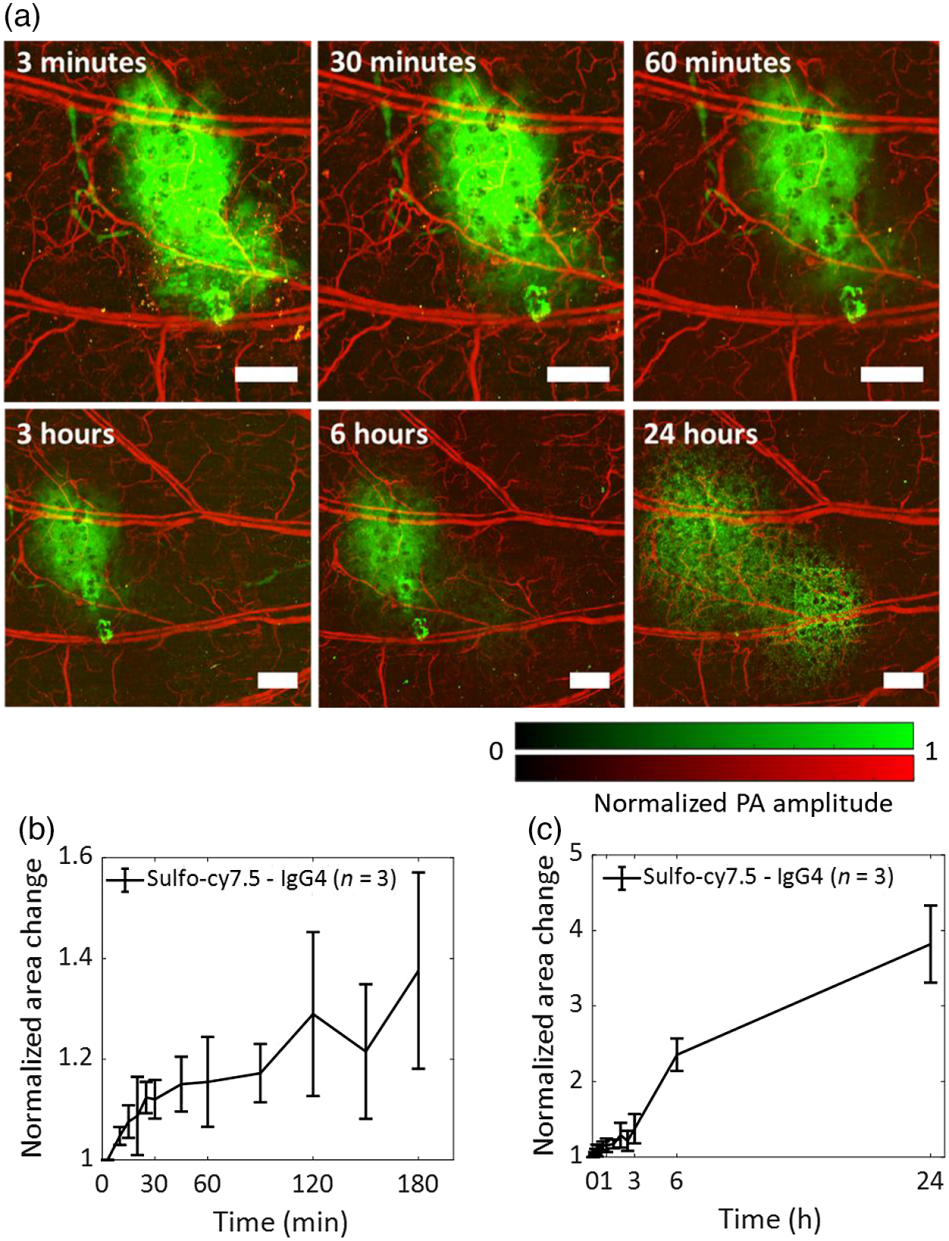
Diffusion of the dye-labeled IgG4 antibody. (a) OR-PAM shows diffusion of the dye-labeled IgG4 antibody (green) along with blood vessels (red) in the mouse ear. The images at 3 h, 6 h, and 24 h are of a larger field of view. (b) The area of the sulfo-cy7.5 dye labeled IgG4 antibody increased by about 40% after the first 3 h after injection. (c) The antibody area increased by about 300% after 24 h of injection. The mice were kept awake in their cages between the 3 h and 6 h, and the 6 h and 24 h time points. All data represent mean ± standard deviation (n=3). PA, photoacoustic. Scale bars, 500  μm.

We studied the antibody movement at the injection site during imaging by estimating the change in the lateral 2D area occupied by the dye-labeled antibody [Fig. 2(a)]. In the first 60 min, the area occupied by the antibody increased by 10% to 15% [Fig. 2(b)]. This is in stark contrast to a smaller size dye-labeled insulin lispro (∼6.8  kDa) in the Humalog formulation, the area of which was reported to increase by 50% to 60% in the first 60 min using the same dye and photoacoustic imaging technique.^21^ After 24 h, the area occupied by the antibody is roughly four times the initial area following injection [Fig. 2(c)]. At 3 h post-injection, when a majority of the PBS is absorbed by the blood vessels [Fig. S2(d) in the Supplemental Material], the antibody is primarily dissolved in the interstitial fluid, and hence, the flow of interstitial fluid may significantly influence the movement of the antibody in addition to other factors, i.e., diffusion and convection.^5^^,^^27^^,^^28^

### 3.2 Measurement of Oxygen Saturation (${\mathrm{sO}}_{2}$) in Local Blood

**Fig. 3:**
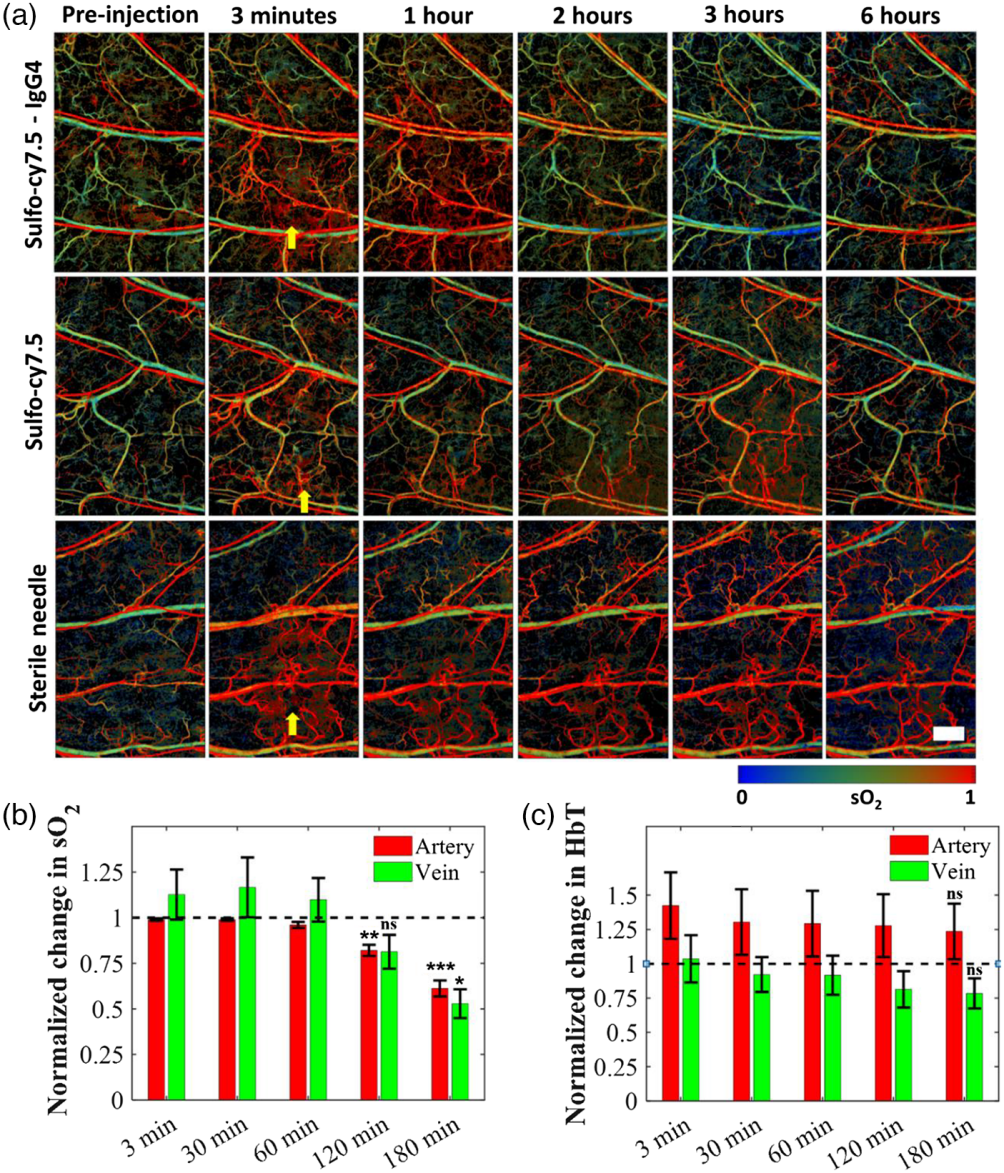
Functional changes in the mouse blood caused by the antibody. (a) Blood ${\mathrm{sO}}_{2}$ starts decreasing after 2 h of injection of the dye-labeled IgG4 antibody. (b) No significant change in blood ${\mathrm{sO}}_{2}$ after injection of the sulfo-cy7.5 dye in PBS. (c) Insertion of sterile needle does not cause any decrease in the blood ${\mathrm{sO}}_{2}$. (d) Statistically significant changes in ${\mathrm{sO}}_{2}$ of major arteries and veins are observed with respect to the pre-injection levels. (e) No significant change in the total hemoglobin (HbT) was observed throughout imaging in the major arteries and veins. The horizontal dashed lines refer to the pre-injection values. Number of mice, n=5; data represent mean ± standard error of the mean. All p values at the mentioned time points were calculated using paired t-test with respect to the pre-injection time points; p>0.05, ns; p<0.05, *; p<0.01, **; p<0.001, ***. Yellow arrows represent points of injections. The mice were kept awake in their cages between the 3 h and 6 h time points. Scale bar, 500  μm.

**Fig. 4:**
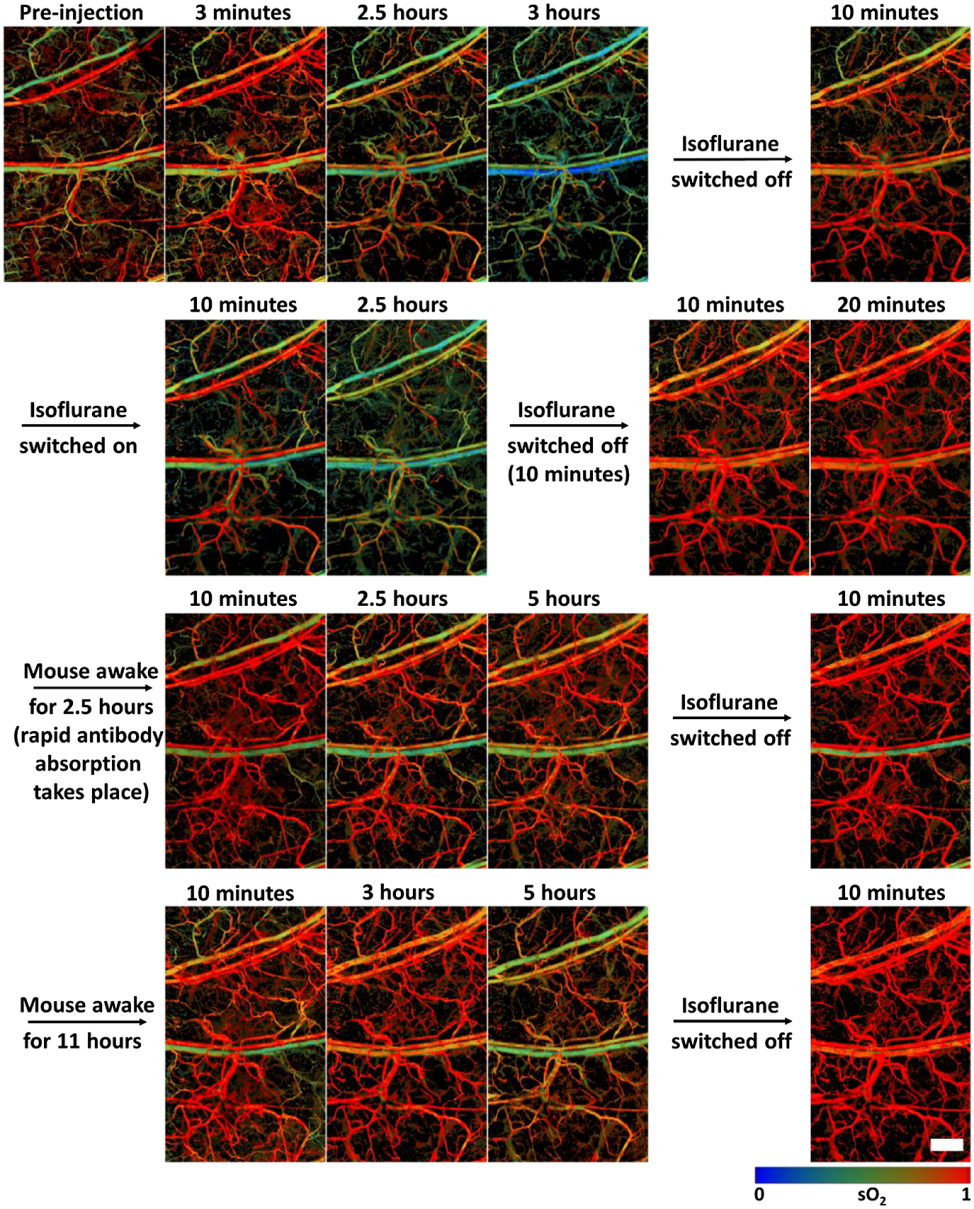
Dependence of blood ${\mathrm{sO}}_{2}$ on isoflurane anesthesia upon injection of dye-labeled IgG4 antibody in a mouse. The time points are reset to zero at every isoflurane on/off activity. Scale bar, 500  μm.

We monitored the effects of the dye-labeled IgG4 isotype control antibody on the local blood ${\mathrm{sO}}_{2}$ using the 532 and 559 nm light through the OR-PAM.^29^ The use of OR-PAM to measure blood oxygen saturation especially in shallow tissues, e.g., mouse ear, has been previously reported.^29^^–^^31^ After subcutaneous injection of the dye-labeled antibody, we initially saw an increase in local blood ${\mathrm{sO}}_{2}$ in veins due to the needle insertion. However, ∼2  h post-injection, the value of ${\mathrm{sO}}_{2}$ in both the veins and arteries dropped [Fig. 3(a)]. After switching off the isoflurane supply, and allowing the mouse to awaken, the ${\mathrm{sO}}_{2}$ returned to the normal physiological levels without the observation of any local or systemic adverse effects on the mouse. Notably, we did not observe any ${\mathrm{sO}}_{2}$ decrease upon injecting sulfo-cy7.5 in PBS [Fig. 3(b)]. However, like the antibody, the injection of sulfo-cy7.5 in PBS instantaneously led to the increase of venous ${\mathrm{sO}}_{2}$ at the point of injection to over 95%, which then gradually decreased over time to return to normal physiological levels.^32^ The insertion of an empty sterile needle produced a similar result; thus, suggesting that the ${\mathrm{sO}}_{2}$ increase is not due to the chemical effects of the formulations or buffers but is likely due to the needle. It is not fully understood what mechanism may result in the sudden and significant rise of local blood ${\mathrm{sO}}_{2}$ levels, which takes a protracted time to return to normal physiological levels in the case of sulfo-cy7.5 or a sterile needle without injection [Fig. 3(c)]. The hypothesis is that the needle may be causing damage to the tissue, leading to transient local tissue hypoxia,^33^^,^^34^ and hence the high ${\mathrm{sO}}_{2}$ blood from arteries is directly passed into the veins. However, in the antibody studies, although the value of local blood ${\mathrm{sO}}_{2}$ decreased after 2 h [Fig. 3(d)], the total local hemoglobin amount remained similar [Fig. 3(e)] for the duration of the experiment, thus, indicating that there is no change in the local blood content. We did not observe any significant change in the local OEF $\left(\frac{{\mathrm{sO}}_{2\text{artery}}-{\mathrm{sO}}_{2\text{vein}}}{{\mathrm{sO}}_{2\text{artery}}}\right)$, indicating that the oxygen consumption of the tissue is unlikely to play a role in the decrease of local blood ${\mathrm{sO}}_{2}$ [Fig. S3(a) in the Supplemental Material]. To exclude the possibility that anesthesia used in the imaging experiments is responsible for this, we imaged a mouse ear for 5 h without performing any injection and observed no changes in the local blood ${\mathrm{sO}}_{2}$ [Fig. S3(b) in the Supplemental Material]. The observed local blood ${\mathrm{sO}}_{2}$ decrease for the unlabeled IgG4 isotype control antibody confirmed that the sulfo-cy7.5 dye labeling is unlikely to play any role [Fig. S3(c) in the Supplemental Material]. To verify if the decrease of local blood ${\mathrm{sO}}_{2}$ is due to the combination of the monoclonal IgG4 antibody and anesthesia, we performed an experiment wherein the isoflurane was switched on and off at regular intervals (Fig. 4). Upon switching off the isoflurane for 10 min after 3 h of anesthesia, we observed an increase in the ${\mathrm{sO}}_{2}$ levels across the whole field of view, which returned to normal physiological levels within the next 10 to 15 min. Upon re-anesthetizing with isoflurane, the ${\mathrm{sO}}_{2}$ decreased again after about 2 to 2.5 h and surged again upon switching off the isoflurane. The mouse was then fully awakened and left to freely roam in the cage (with food and water) for around 2 to 2.5 h. The muscle movement in the ears during this period is believed to facilitate lymphatic absorption of the antibody. Upon re-anesthetizing and re-imaging the same mouse, the ${\mathrm{sO}}_{2}$ decreased to a lesser extent, over a longer period, requiring up to 5 h as opposed to 2 to 2.5 h, and rose again after switching off the isoflurane. We observed a similar pattern upon keeping the mouse awake in its cage for 10 h. Although the changes in ${\mathrm{sO}}_{2}$ were striking, we did not observe any acute adverse effects or discomfort in any of the mice that were injected with the labeled antibody. To confirm that the blood ${\mathrm{sO}}_{2}$ decrease is not specific to only one type of anesthesia, i.e., isoflurane gas initially used in this study, we monitored and observed a similar decrease in the blood ${\mathrm{sO}}_{2}$ levels in the mouse ear after injection of the dye-labeled IgG4 antibody under ketamine induced anesthesia (Fig. S4 in the Supplemental Material).

## 4 Conclusions

Through pharmacokinetic experiments of dye-labeled and unlabeled antibody solutions, we showed that the extent of dye labeling in these experiments did not affect the absorption characteristics of the antibody and that subcutaneous administration in the mouse ear was suitable for studying the subcutaneous behavior of the monoclonal IgG4 antibody. Our experiments proved that light-based optical absorption techniques cannot quantify injection site absorption of large-sized molecules, i.e., antibodies, unless the dynamic change in the molar absorption coefficient is deduced. We also quantified antibody movement at the injection site caused by interstitial fluid flow. Through a series of experiments, we showed that the monoclonal human IgG4 isotype control antibody locally reduced oxygen saturation in mouse blood after prolonged anesthesia and that oxygenation was recovered within a few minutes after switching off the isoflurane. The underlying mechanisms that lead to a decrease in oxygenation in anesthetized mice are not well-understood at this point, and further experiments are required; however, this is beyond the scope of this work. Given the widespread preclinical and clinical use of antibodies at an exponentially increasing rate, these results open new avenues of investigation and call for further studies focused on the injection site characteristics and effects of monoclonal antibodies.

## Data Availability

The data that support the conclusions of this paper are mentioned in the main text or the supplementary information.
