# Supplementary material for: Photoacoustic imaging of the dynamics of a dye-labeled IgG4 monoclonal antibody in subcutaneous tissue reveals a transient decrease in murine blood oxygenation under anesthesia
Source: J Biomed Opt. 2023 Nov 30;28(11):116002. doi: 10.1117/1.JBO.28.11.116002 (PMC10704085; doi:10.1117/1.JBO.28.11.116002)
Supplement: Supplementary file 1 [file JBO_028_116002_SD001.docx]

**Supplementary information**

**Photoacoustic imaging of the dynamics of a dye-labeled IgG4 monoclonal antibody in subcutaneous tissue reveals a transient decrease in murine blood oxygenation under anesthesia**

**Anjul Khadria^1^, Chad D. Paavola^2^, Konstantin Maslov^1^, Patricia L. Brown-Augsburger^2^, Patrick F. Grealish^2^, Emmanuel Lozano^2^, Ross L. Blankenship^2^, Rui Cao^1^, Junhui Shi^1^, John M. Beals^3^*, Sunday S. Oladipupo^2^*, Lihong V. Wang^1,4^***

^1^Caltech Optical Imaging Laboratory, Andrew and Peggy Cherng Department of Medical Engineering, California Institute of Technology, Pasadena, California, 91125, USA

^2^Lilly Research Laboratories, Eli Lilly and Company, Lilly Corporate Center, Indianapolis, Indiana, 46285, USA

^3^Lilly Research Laboratories, Eli Lilly and Company, Lilly Biotechnology Center, San Diego, California, 92121, USA

^4^Caltech Optical Imaging Laboratory, Department of Electrical Engineering, California Institute of Technology, Pasadena, California, 91125, USA

**Figure S1:** Molar absorption spectra of the dye-labeled IgG4 antibody, deoxygenated hemoglobin and oxygenated hemoglobin.


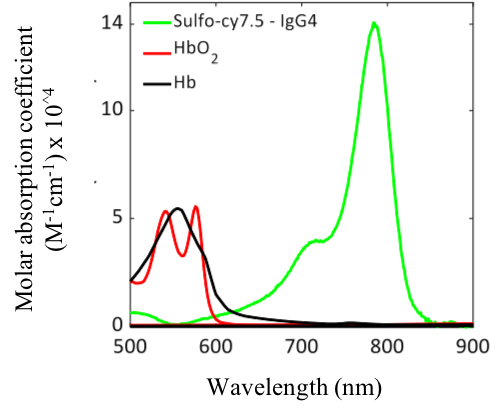


**Figure S2: Quantification.** (a) Pharmacokinetics data of unlabeled IgG4 isotype control antibody in mice following IV and subcutaneous administration in the torso or ear; data represent mean ± standard deviation. (b, c) Photoacoustic quantification of absorption of dye-labeled IgG4 antibody for 24 hours and 6 hours. (d) Photoacoustic quantification of sulfo-cy7.5 dye dissolved in PBS buffer; data represent mean ± standard error of the mean.

**a**

**b**

**c**

**d**


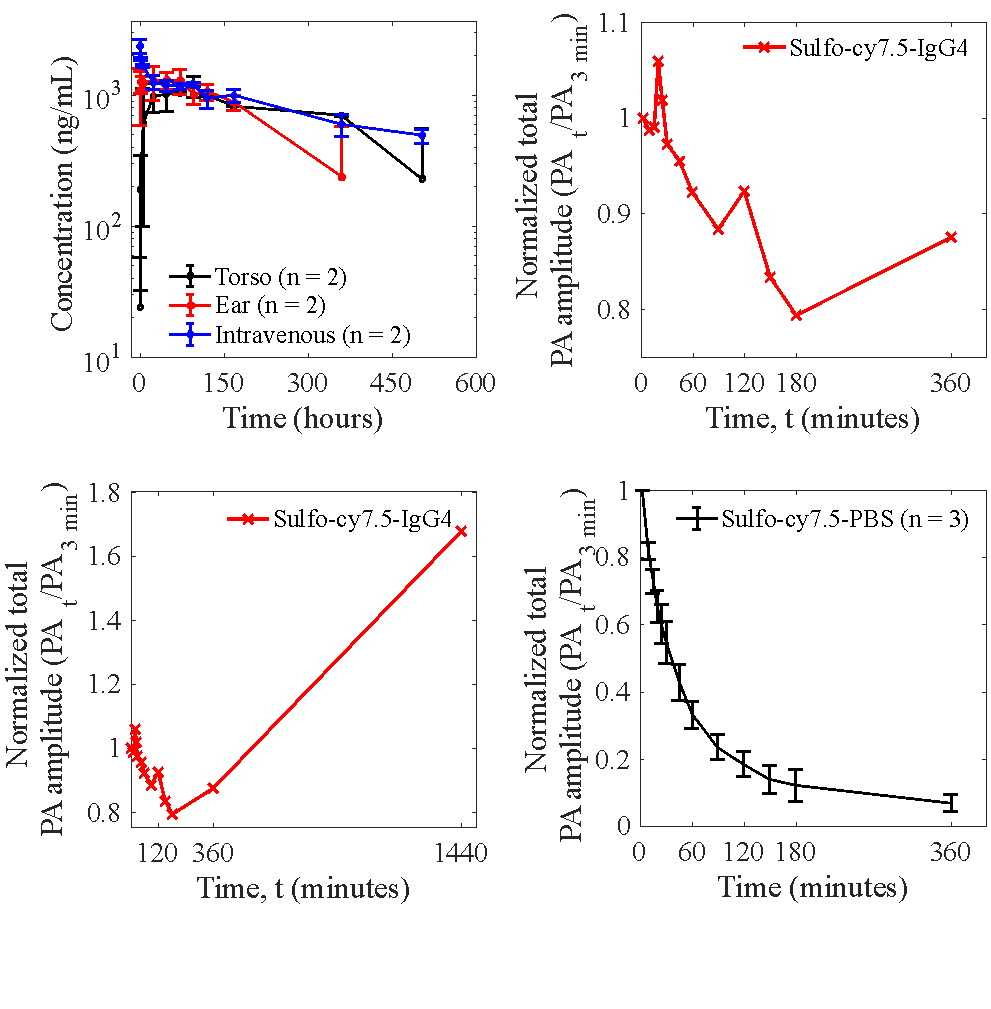


**Table S1: Mean pharmacokinetic parameters of unlabeled IgG4 antibody**

|  | **t_1/2_**  **(h)** | **T_max_**  **(h)** | **C_max_**  **(ng/mL)** | **AUC_0-168_**  **(h*μg/mL)** | **AUC_all_**  **(h*μg/mL)** | **AUC_INF_obs_**  **(h*μg/mL)** | **Cl_obs**  **(mL/h/kg)** |
| --- | --- | --- | --- | --- | --- | --- | --- |
| **IV-tail** | 304 | 1 | 2351 | 195 | 414 | 629 | 0.26 |
| **SC-torso** | 520 | 96 | 1179 | 160 | 408 | 867 | 0.20 |
| **SC-ear** | 511 | 48 | 1355 | 197 | 330 | 944 | 0.19 |

**Table S2: Mean pharmacokinetic parameters of sulfo-cy7.5 dye-labeled IgG4 antibody**

|  | **t_1/2_**  **(h)** | **T_max_**  **(h)** | **C_max_**  **(ng/mL)** | **AUC_0-168_**  **(h*μg/mL)** | **AUC_all_**  **(h*μg/mL)** | **AUC_INF_obs_**  **(h*μg/mL)** | **Cl_obs**  **(mL/h/kg)** |
| --- | --- | --- | --- | --- | --- | --- | --- |
| **IV-tail** | 238 | 1 | 2233 | 145 | 288 | 379 | 0.40 |
| **SC-torso** | 117 | 36 | 1206 | 130 | 158 | 235 | 0.70 |
| **SC-ear** | 444 | 12 | 1808 | 199 | 405 | 1005 | 0.30 |


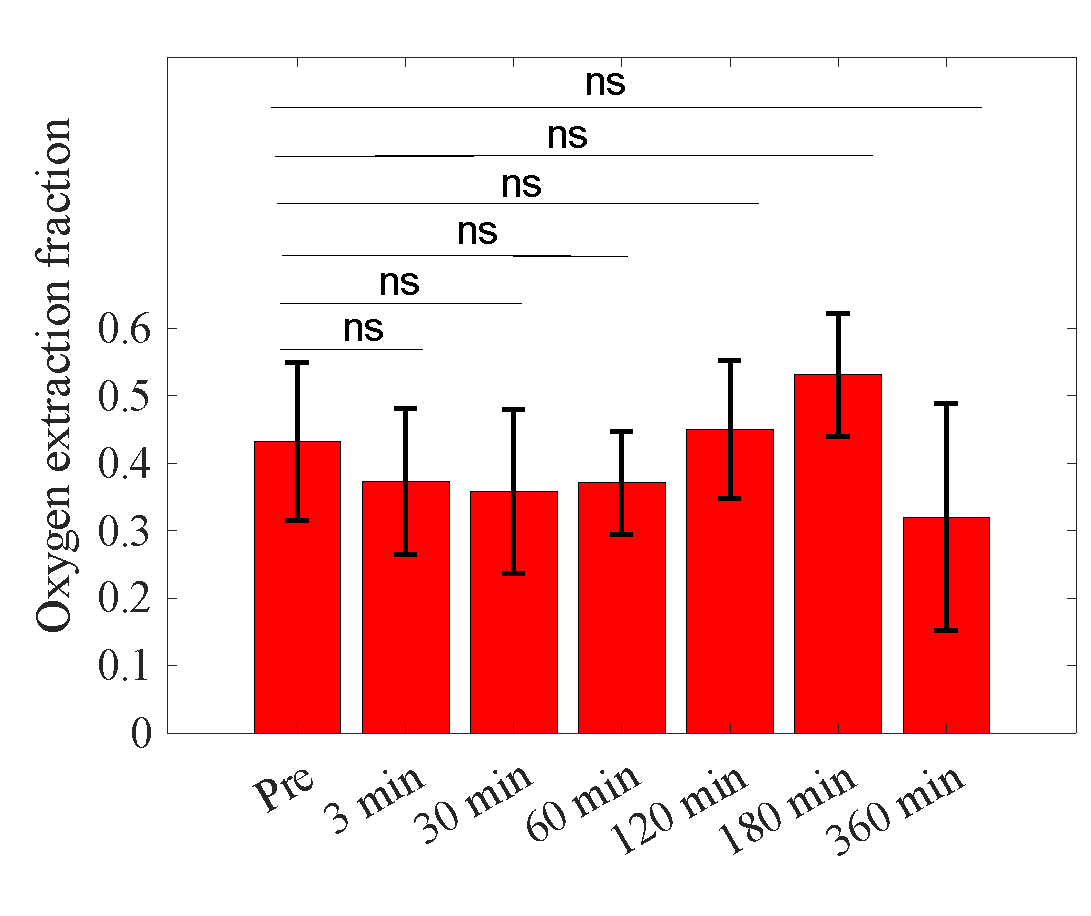

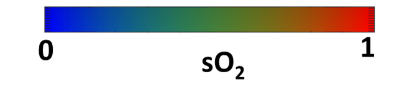

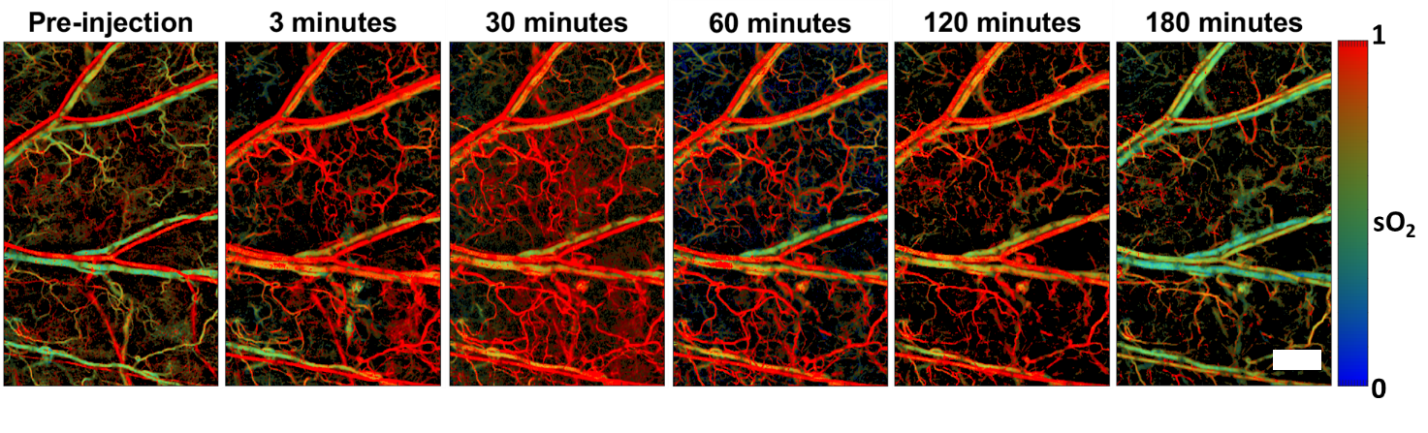


**Figure S3: Functional changes caused by the human IgG4 antibody.** (a) No significant change cuased by the dye-labeled antibody in the oxygen extraction factor with respect to the preinjection value. Number of mice, n = 5; data represent mean ± standard deviation. All p values at the mentioned time points were calculated using paired t-test with respect to the pre-injection time point; p > 0.05, ns. (b) No change of blood sO_2_ in mouse ear after being kept under anesthesia for 5 hours continuously. (c) Blood sO_2_ in the mouse ear decreases after injection of unlabeled human IgG4 antibody. Scale bars, 500 μm.

**a**

**b**


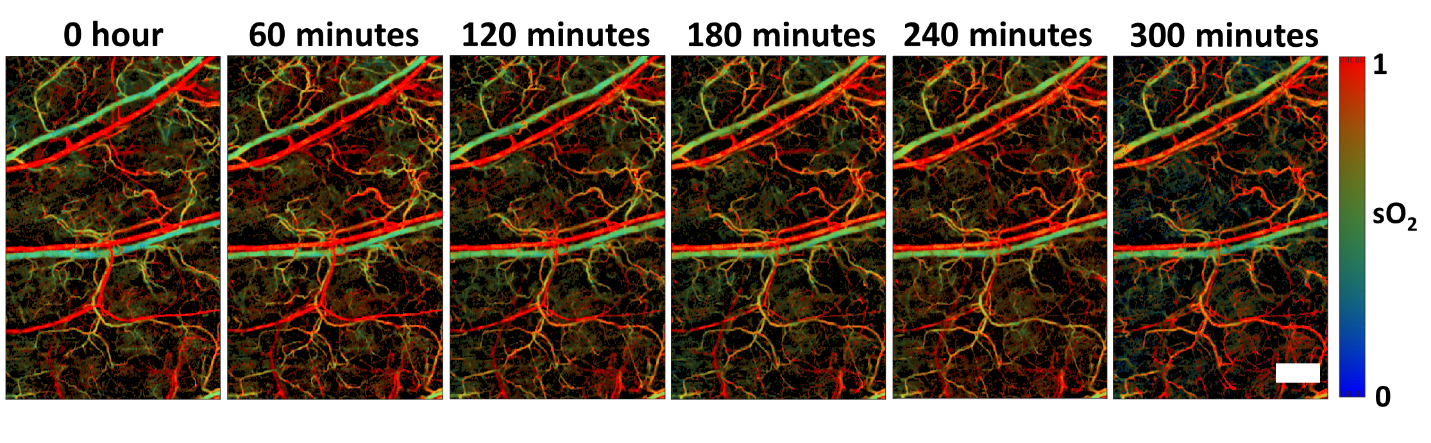


**c**

**
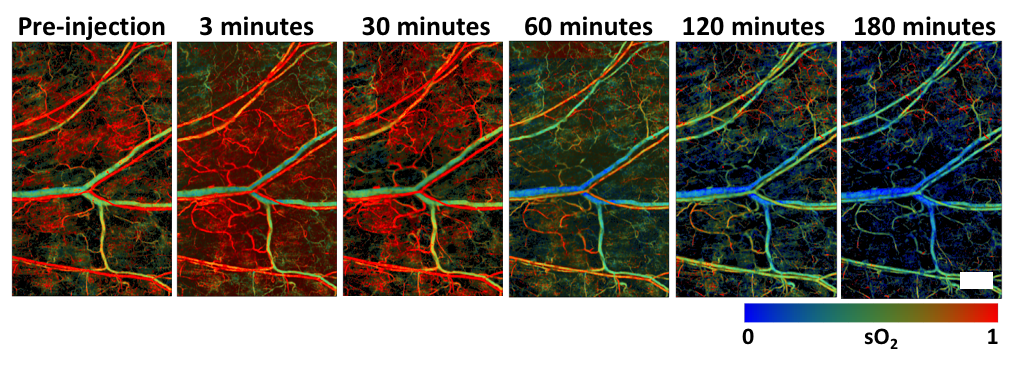
**

**Figure S4: Functional changes under ketamine/xylazine anesthesia.** Blood sO_2_ in the mouse ear decreases after injection of dye-labeled human IgG4 antibody while the mouse was under ketamine/xylazine anesthesia. Decrease of blood sO_2_ in the mouse ear seen in the major vein even at 1 hour post injection. Scale bar, 500 μm.
